# Taxonomic treatment of three Thai *Garcinia* species (section *Brindonia*, Clusiaceae), with six lectotypifications and conservation status assessments

**DOI:** 10.3897/phytokeys.275.188257

**Published:** 2026-05-19

**Authors:** Chatchai Ngernsaengsaruay

**Affiliations:** 1 Department of Botany, Faculty of Science, Kasetsart University, Chatuchak, Bangkok 10900, Thailand Biodiversity Center, Kasetsart University (BDCKU) Bangkok Thailand; 2 Biodiversity Center, Kasetsart University (BDCKU), Chatuchak, Bangkok 10900, Thailand Department of Botany, Faculty of Science, Kasetsart University Bangkok Thailand

**Keywords:** Dioecy, *Garcinia bancana*, *Garcinia costata*, *Garcinia parvifolia*, Guttiferae, Malpighiales, taxonomy

## Abstract

The taxonomy of three *Garcinia* species—*G. bancana*, *G. costata*, and *G. parvifolia* (section *Brindonia*, Clusiaceae)—is revised for Thailand. Morphological descriptions, illustrations, and photographs are provided, along with notes on distribution, phenology, conservation status, etymology, vernacular names, uses, specimens examined, habitats, and ecology. All three species are restricted to Peninsular Thailand and produce edible fruits. Six names are lectotypified here, including *G. bancana* and its three synonyms (*G. lamponga*, *G. leucandra*, and *G. oxyedra*), as well as *G. parvifolia* and its synonym (*G. globulosa*). *Garcinia costata* is assessed as Vulnerable (VU) under IUCN criterion D1, whereas *G. bancana* and *G. parvifolia* are assessed as Least Concern (LC).

## Introduction

*Garcinia* sect. *Brindonia* (Thouars) Choisy, recently updated by Gaudeul et al. (2024) to include 91 recognized species, is the largest section of the genus. Species in this section are distributed in Madagascar, Indomalaya, tropical Australasia, and Oceania (Gaudeul et al. 2024). The section is characterized by flowers with four sepals and four petals. Staminate flowers in most species of the section usually lack a pistillode. However, in some species (e.g., *G. atroviridis* Griff. ex T. Anderson, *G. pedunculata* Roxb. ex Buch.-Ham., and *G. sopsopia* (Buch.-Ham.) Mabb.), the pistillode is consistently present. In contrast, in certain species (e.g., *G. cowa* Roxb. ex Choisy var. cowa and *G. oliveri* Pierre), the pistillode is variable, being either present or absent. Stamens are united into a single central bundle or into a ring when a pistillode is present, as in *G. atroviridis*. Anthers are 4-thecous, rarely 2-thecous. Ovaries are multilocular. Stigmas are divided into as many distinct rays as there are ovary locules and are usually papillate. In many species, the fruits have furrows or grooves along the septal radii. Inflorescences are terminal or axillary, bearing one to many flowers (Jones 1980; Ngernsaengsaruay 2022; Gaudeul et al. 2024; Ngernsaengsaruay et al. 2025a; Ngernsaengsaruay and Chanton 2026). The fruits and leaves of many species in this section are edible, including *G. atroviridis*, *G. cowa* var. cowa, *G. lanceifolia* Roxb., *G. pedunculata*, *G. schomburgkiana* Pierre, *G. siripatanadilokii* Ngerns., Meeprom, Boonth., Chamch. & Sinbumr., and *G. sopsopia* (Buch.-Ham.) Mabb. (Ngernsaengsaruay 2022; Ngernsaengsaruay et al. 2022b, 2025a; Ngernsaengsaruay and Chanton 2026).

In Thailand, the genus *Garcinia* was first enumerated by Craib (1925), who recognized 20 species. Gardner et al. (2000) reported six species from northern Thailand and later documented 23 species, including five unidentified species, from Peninsular Thailand (Gardner et al. 2015). A taxonomic revision of *Garcinia* in Thailand has recently been undertaken by the author as part of the Flora of Thailand project, and treatments of several sections have since been published (Ngernsaengsaruay and Suddee 2016, 2022; Ngernsaengsaruay 2022; Ngernsaengsaruay and Chanton 2024, 2026; Ngernsaengsaruay et al. 2022a, 2022b, 2023a, 2023b, 2024a, 2024b, 2025a, 2025b).

Based on previous and current publications, approximately 11 accepted species of *Garcinia* sect. *Brindonia* have been reported from Thailand. However, most identifications have relied primarily on published literature due to the lack of a comprehensive taxonomic revision for Thailand. This paper provides a taxonomic treatment of three species of *Garcinia* sect. *Brindonia* occurring in Thailand: *G. bancana* Miq., *G. costata* Hemsl. ex King, and *G. parvifolia* (Miq.) Miq. This treatment includes lectotypifications, morphological descriptions, and illustrations, together with notes on distribution, phenology, conservation status, etymology, vernacular names, uses, specimens examined, habitats, and ecology.

## Materials and methods

Collected specimens were examined by consulting relevant literature (e.g., Miquel 1861, 1864; Anderson 1874; Pierre 1883; King 1890; Vesque 1889, 1893; Ridley 1910, 1922; Corner 1939, 1952; Backer and Bakhuizen van den Brink 1963; Kochummen and Whitmore 1973; Whitmore 1973; Jones 1980; Keng 1990; Gardner et al. 2015) and through comparison with herbarium specimens housed in the following herbaria: AAU, BK, BKF, BM, C, CMUB, K, P, PSU, and SING. Additional specimens were examined via the digital herbarium databases of BO, CAL, E, L, MEL, and U, as well as through the Global Biodiversity Information Facility (GBIF, https://www.gbif.org/) and JSTOR (https://www.jstor.org/). Herbarium acronyms follow Thiers (2026). All cited specimens were examined by the author unless otherwise stated. The taxonomic history of the species was compiled from the literature and online databases (IPNI 2026; POWO 2026; WFO 2026). Morphological characters, distribution, habitat, ecology, phenology, and uses were documented based on historical and newly collected herbarium specimens, as well as the author’s field observations. Vernacular names were compiled from examined specimens and relevant literature (e.g., Pooma and Suddee 2014). Thailand’s floristic regions follow *Flora of Thailand* Vol. 16(4) (The Forest Herbarium, Department of National Parks, Wildlife and Plant Conservation 2025). Conservation status assessments were conducted in accordance with the IUCN Red List Categories and Criteria (IUCN Standards and Petitions Committee 2024), incorporating GeoCAT analyses (Bachman et al. 2011) and field data.

## Results and discussion

### Taxonomic treatment

#### Garcinia bancana

Taxon classificationPlantaeMalpighialesGuttiferae

Miq., Fl. Ned. Ind., Eerste Bijv. 3: 494. 1861.

79D8A411-643E-54E1-BE3D-BC651F5549FE

Figs 1, 2A

Garcinia bancana Miq., Fl. Ned. Ind., Eerste Bijv. 3: 494. 1861 et Ann. Mus. Bot. Lugduno-Batavi 1(7): 208. 1864; T. Anderson in Hook. f., Fl. Brit. India 1(2): 263. 1874; Pierre, Fl. Forest. Cochinch. 1(5): 26, t. 81D. 1883; King, J. Asiat. Soc. Bengal, Pt. 2, Nat. Hist. 59(2): 162. 1890; Vesque, Epharmosis 2: 21, t. 137. 1889 et in A. DC. & C. DC., Monogr. Phan. 8: 458. 1893; Ridl., Fl. Malay Penins. 1: 174. 1922; Corner, Wayside Trees Mal. 1: 314, fig. 103. ed. 2. 1952; Kochummen & Whitmore, Gard. Bull. Singapore 26(2): 272. 1973; Whitmore in Whitmore, Tree Fl. Malaya 2: 206. 1973; S. W. Jones, Morphology and Major Taxonomy of Garcinia (Guttiferae), Ph.D. Thesis (unpublished): 329. 1980; H. Keng, Concise Fl. Singapore: 48. 1990; Phengklai et al., Fl. Peat Swamp Areas of Narathiwat: 50, 197. 1991; I. M. Turner, Gard. Bull. Singapore 47(1): 260. 1995; S. Gardner et al., Forest Trees S. Thailand 1 (A–Es): 349, fig. 536. 2015. Type. Indonesia, Sumatra, Bangka, s.d., *J. E. Teijsmann s.n*. (lectotype: designated here, K! [K000677726] (Fig. 2A); isolectotypes: BM digital image! [BM000611762], L digital image! [L2408723], P! [P04663162, P04663163], U digital image! [U1208244]).
*= Garcinia oxyedra* Miq., Fl. Ned. Ind., Eerste Bijv. 3: 494. 1861 et Ann. Mus. Bot. Lugduno-Batavi 1(7): 208. 1864. Type. Indonesia, Sumatra, Siboga, s.d., *J. E. Teijsmann HB643* (lectotype: designated here, U digital image! [U0123401]; isolectotype: P! [P04700617]).
*= Garcinia lamponga* Miq., Fl. Ned. Ind., Eerste Bijv. 3: 494. 1861 et Ann. Mus. Bot. Lugduno-Batavi 1(7): 208. 1864. Type. Indonesia, Sumatra, Lampong, Maranga, s.d., *J. E. Teijsmann HB4456* (lectotype: designated here, U digital image! [U0104026]; isolectotype: L digital image! [L2408730]).
*= Garcinia leucandra* Pierre, Fl. Forest. Cochinch. 1(5): 27, t. 88A, B. 1883; Vesque, Epharmosis 2: 21, t. 138. 1889. Type. Indonesia, Sumatra, Nov 1881, Treub (*Herb. L. Pierre 4166*) (lectotype: designated here, P! [P04700362]; isolectotype: P! [P04700360]).

##### Description.

***Habit*** evergreen trees, dioecious, 7–20(–25) m tall, 25–135 cm GBH; exudate yellow and sticky; branches decussate, horizontal or nearly horizontal; young branchlets green, 4-angular, glabrous. ***Bark*** dark brown or dark grayish brown, smooth or scaly; inner bark pinkish, brownish red, or red. ***Terminal bud*** concealed between the bases of the uppermost pair of petioles. ***Leaves*** decussate; lamina elliptic, obovate, or oblanceolate-obovate, 10–23 × 4–11.5 cm, apex acute, rounded, retuse, or emarginate, base cuneate, margin entire or repand, subcoriaceous, glossy dark green above, paler below, glabrous on both surfaces, midrib slightly raised (proximal part) and flattened (distal part) above, raised below, secondary veins 16–20 on each side, 4–6.5 mm apart from each other, departing from the midrib at an angle of 35°–45°, curving towards the margin and connected in distinct loops and united into an intramarginal vein, flattened above, slightly raised below, with intersecondary veins, veinlets reticulate, visible above, faint below, interrupted long wavy glandular lines (which are exudate containing canals), of differing lengths, running across the secondary veins to the apex, visible below; petiole green, 1–3.2 cm long, 2–7 mm diam., grooved above, slightly transversely rugose, glabrous, with a basal appendage clasping the branchlet; fresh leaves brittle when crushed. ***Inflorescences*** axillary, borne on short, leafless lateral branchlets, in fascicles of 2–12-flowered cymes (staminate inflorescence usually bearing more flowers than pistillate ones), or sometimes a solitary flower (in pistillates). ***Flowers*** unisexual, 4-merous; pedicel pale green to greenish yellow or yellow, glabrous; bracteoles broadly triangular, 1.5–3 mm long; sepals and petals decussate, concave, slightly thick and fleshy, glabrous; sepals pale yellow to yellow; petals pale yellow to yellow or creamy white. ***Flower buds*** pale green, becoming greenish yellow to yellow before anthesis, subglobose or globose, 2–5 mm diam. ***Staminate flowers*** 4–7 mm diam., usually smaller than pistillate ones; pedicel 4–8 mm long, 0.8–1.5 mm diam.; sepals 4, broadly elliptic, elliptic, suborbicular, or orbicular, 3–5 × 2–4 mm, the outer pair slightly larger than the inner pair, apex rounded; petals 4, broadly elliptic or elliptic, 3–5 × 2–3.5 mm, subequal, apex rounded; stamens numerous, united into a single central 4-sided bundle; filaments very short; anthers small, 4-thecous, longitudinally dehiscent; pistillode absent. ***Pistillate flowers*** 0.7–1 cm diam.; pedicel 3.5–6 mm long, 2–3 mm diam., usually shorter and thicker than staminate ones; sepals and petals same as in staminate flowers; staminodes 6–10, free or united into two or three bundles, surrounding the ovary; pistil fungiform; ovary pale green, subglobose or globose; stigma pale yellow, convex, unlobed, rough. ***Fruits*** berries, green turning yellow to orangish yellow when ripe, smooth and glabrous, cut fruits with a sticky yellow exudate, globose or subglobose, 3–5 × 2.8–5.5 cm, unlobed, apex concave or not, base concave, pericarp fleshy, 5–8 mm thick, drying dark brownish black, irregularly shrunken, coarsely wrinkled; persistent stigma sunken or not, 2.5–4.5 mm diam., unlobed, rough; persistent sepals slightly larger than in flowering material; fruiting stalk short and thick, 2–5 mm long, 2.5–4 mm diam. ***Seeds*** 8–12, sometimes aborted (1–3), semi-ellipsoid, 1–2 × 0.6–1 cm, rounded at both ends, with a yellow to orangish yellow fleshy pulp.

**Figure 1.**
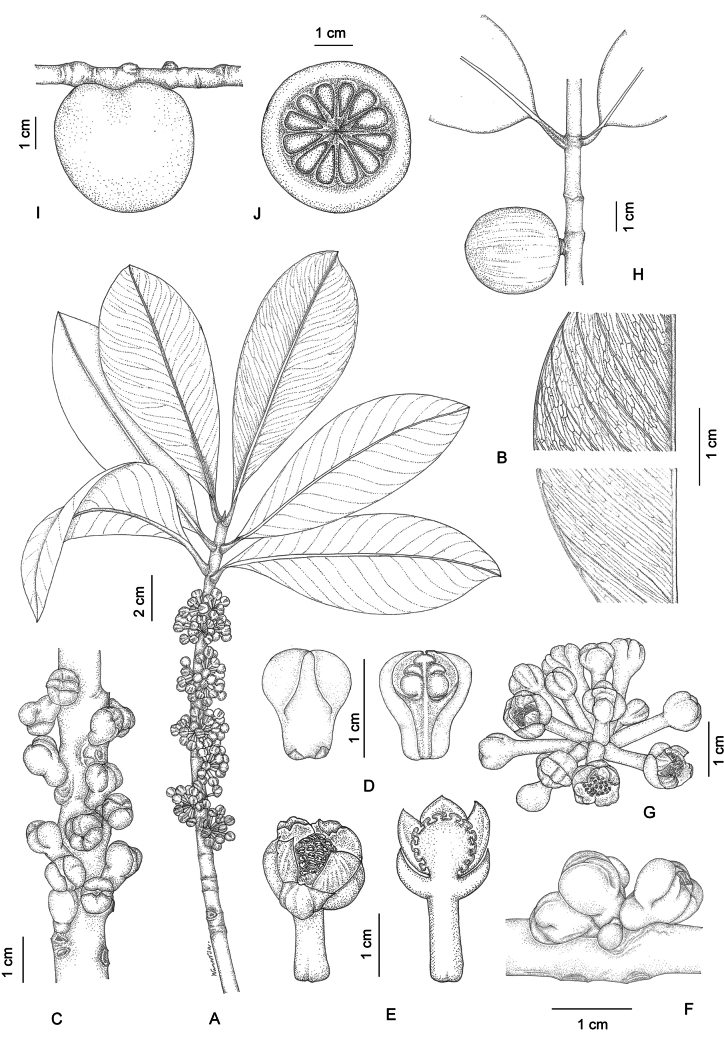
*Garcinia bancana*. **A**. Branchlets with leaves and inflorescences bearing staminate flower buds; **B**. Part of the abaxial surface of the leaf; **C, F**. Branchlets with inflorescences bearing open pistillate flowers and flower buds; **D**. Pistillate flowers; **E**. Staminate flowers; **G**. Inflorescence with open staminate flowers and flower buds; **H**. Branchlets with leaves and fruit; **I**. Branchlets with fruit; **J**. Transverse section of fruit showing seeds. Photo: drawn by Wanwisa Bhuchaisri.

**Figure 2.**
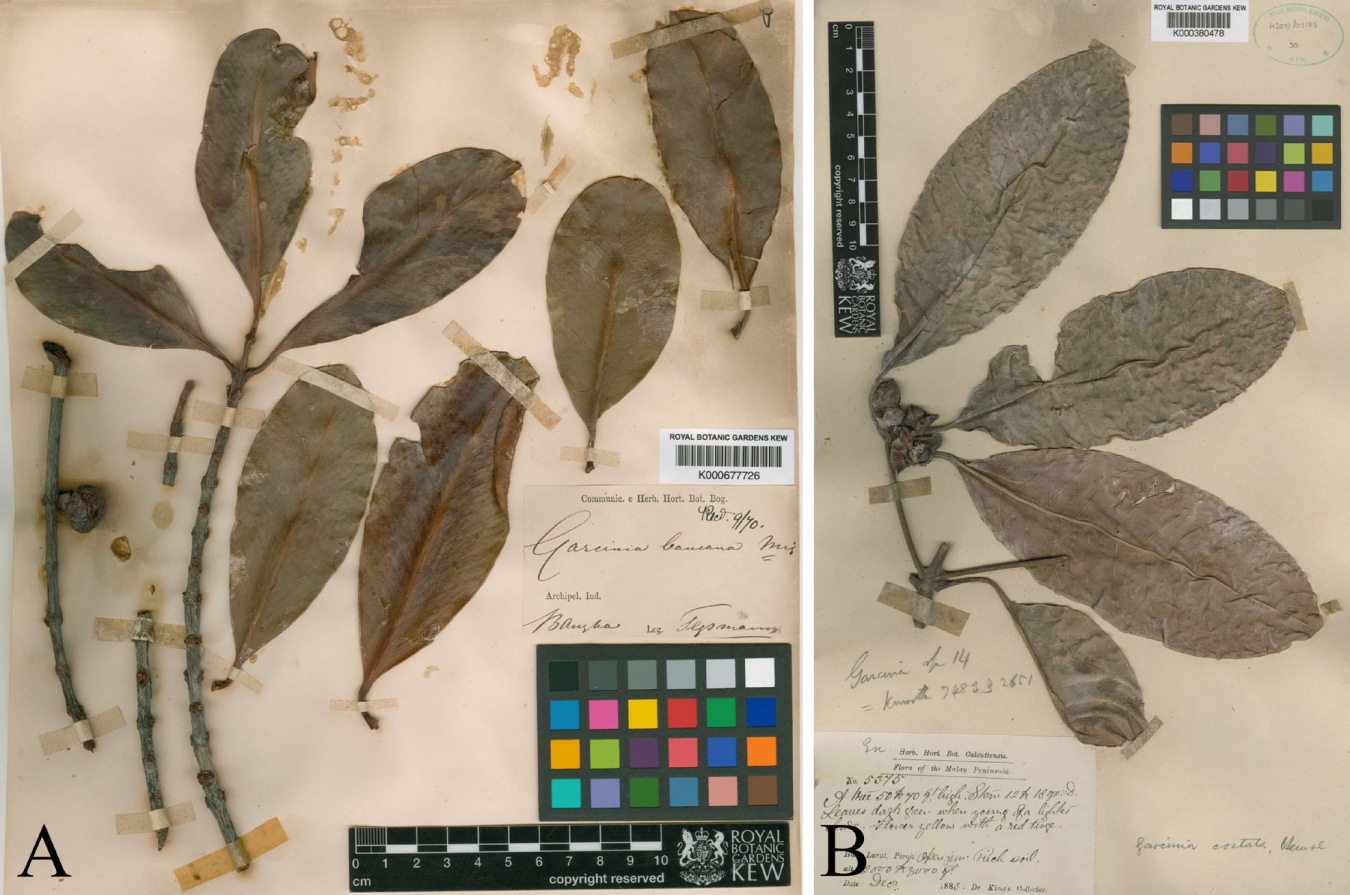
**A**. Lectotype of *Garcinia bancana*, *J. E. Teijsmann s.n*. (K [K000677726]) from Bangka, Sumatra, Indonesia, designated here; **B**. Lectotype of *Garcinia costata*, *G. King’s Collector 5375* (K [K000380478]) from Maxwell’s Hill, Larut, Perak, Peninsular Malaysia, designated by Nazre et al. (2018). Photos: © Board of Trustees of the Royal Botanic Gardens, Kew.

##### Distribution.

**Figure 3.**
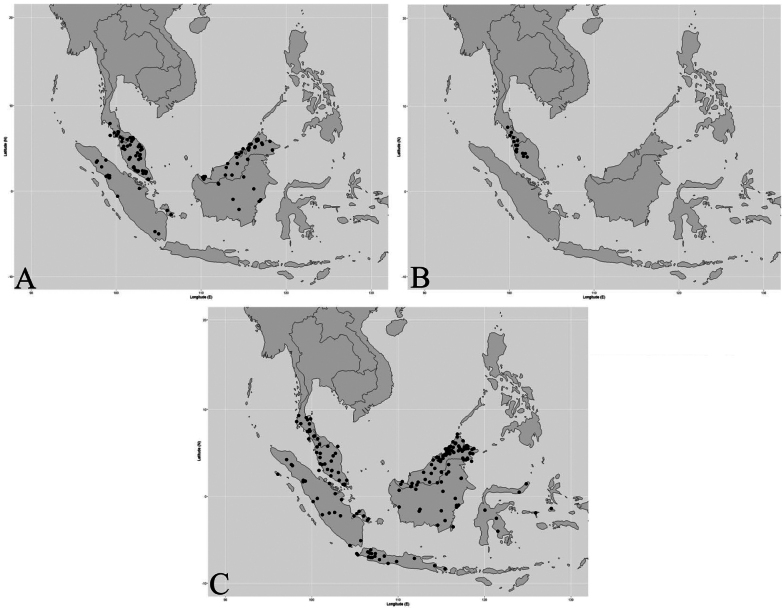
Distribution of three Thai *Garcinia* species. **A**. *Garcinia bancana* is distributed from Peninsular Thailand south to Peninsular Malaysia and Borneo; **B**. *Garcinia costata* is distributed from Peninsular Thailand to Peninsular Malaysia; **C**. *Garcinia parvifolia* is distributed from Peninsular Thailand southwards through Peninsular Malaysia to Borneo and New Guinea. Maps: Pichet Chanton and Chatchai Ngernsaengsaruay.

Peninsular Thailand, Peninsular Malaysia (Kedah, Perak, Pahang, Terengganu, Negeri Sembilan, Malacca, and Johor), Singapore, Indonesia (Sumatra), and Borneo (Sabah, Sarawak, Brunei, East Kalimantan, West Kalimantan, and Central Kalimantan) (Fig. 3A).

##### Distribution in Thailand.

**Peninsular**: Surat Thani, Krabi, Satun, Songkhla, Yala, Narathiwat (Fig. 3A).

##### Habitat and ecology.

The species occurs in tropical lowland evergreen rainforests, often along streams and in peat swamp forests, at elevations from near sea level to 300 m a.s.l.

##### Phenology.

Flowering from February to June; fruiting from March to December.

##### Conservation status.

*Garcinia bancana* has a wide geographic distribution extending from Peninsular Thailand to Borneo and is known from many localities. The species has a large Extent of Occurrence (EOO) of 4,404,899.27 km^2^ and an Area of Occupancy (AOO) of 360 km^2^. In Thailand, it is restricted to the Peninsular region, with an EOO of 30,345.71 km^2^ and an AOO of 44 km^2^. Given its broad distribution, occurrence in many localities, and the absence of evidence of significant threats to the species or its habitats, *G. bancana* is here assessed as Least Concern (LC), following Oldfield (2021).

##### Etymology.

The specific epithet *bancana* refers to Bangka, the type locality of the species (Miquel 1861), an island east of Sumatra, Indonesia, also spelled Banka or Banca.

##### Vernacular names.

Ka-ni-hu-tae (กานิฮูแต) (Malay-Narathiwat, reported by Pooma and Suddee 2014), Ka-ni-hu-ton (กานีฮูตัน) (Malay-Narathiwat, from the specimen *C. Niyomdham 576*); Cha muang ka (ชะมวงกา) (from the specimen *A. Premrasmi 15*), **Cha muang pa** (ชะมวงป่า) (Narathiwat, reported by Pooma and Suddee 2014); Som muang chang (ส้มมวงช้าง) (Satun, Yala, from the specimens *A. F. G. Kerr 14430* and *Unknown 2* (BKF [50346]); Banca mangosteen, Chepurah, Chempurah (Corner 1952); Manggis hutan (Oldfield 2021).

##### Uses.

The fruits are edible and sour. In Peninsular Thailand, the wood is locally used for indoor construction (Phengklai et al. 1991; Niyomdham 1997). In southern Sumatra, the fruits are eaten, and in Sumatra, the timber appears to be valuable and much sought after (Burkill et al. 1966).

##### Lectotypifications.

*Garcinia bancana* was described by Miquel (1861), who cited collections by *Teysmann* (T.) from “Bangka” but did not indicate the herbaria in which the specimens were deposited. Miquel (1811–1871) served as professor of botany and director of the Amsterdam Botanical Garden (1846–1859), the Utrecht Botanical Garden (1859–1871), and, from 1862, the Leiden Rijksherbarium. His private herbarium, which included numerous type specimens, later formed the basis of the general herbarium at U. Additional material described by Miquel, often based on loaned specimens, is housed at L, G, P, and K. Specimens labeled “Ex Herbario Miquel” were generally collected by other botanists (Stafleu and Cowan 1981). Nine sheets of *Teijsmann s.n*. from “Bangka” were located at BM [BM000611762], K [K000677725, K000677726], L [L2408722, L2408723], MEL [MEL2436029, MEL2436030], P [P04663162, P04663163], and U [U0105734, U1208244]. In accordance with Art. 9.6 of the ICN (Turland et al. 2018), these specimens constitute syntypes. Among them, the specimen at K [K000677726] is the most complete and clearly exhibits the diagnostic characters of the species. It is therefore designated here as the lectotype, in accordance with Arts. 9.3 and 9.12 of the ICN (Turland et al. 2018).

*Garcinia oxyedra* was described by Miquel (1861), who cited two gatherings: *T.* (*Teysmann*) from “littore prope Siboga” and *D.* (*Diepenhorst*) from “Priaman,” but did not indicate the herbaria in which the specimens were deposited. Specimens of *Teijsmann HB643* (labeled “*Teysmann 643*” or “*643HB*”) collected from Siboga (also spelled Sibolga) at P [P04700617] and U [U0123401], and Diepenhorst HB640 (labeled “*Scheffer 640HB*”) collected from Priaman (also spelled Pariaman) was located at P [P04700618]. In accordance with Art. 9.6 of the ICN (Turland et al. 2018), these specimens constitute syntypes. Among them, the specimen *Teijsmann HB643* at U [U0123401], housed in Miquel’s private herbarium, is designated here as the lectotype, in accordance with Arts. 9.3 and 9.12 of the ICN (Turland et al. 2018).

*Garcinia lamponga* was described by Miquel (1861), who cited collections by *Teysmann* (*T.*) from “Lampong, prope Maranga” but did not indicate the herbaria in which the specimens were deposited. Two sheets of *Teijsmann HB4456* (labeled “*4456HB*” or “*HB4456*”), collected from Maranga, Lampong (now Lampung Province), were located at L [L2408730] and U [U0104026], and one sheet of *Teijsmann HB4385* (labeled “*4385HB*”), collected from the same locality, at U [U0002398]. In accordance with Art. 9.6 of the ICN (Turland et al. 2018), these specimens constitute syntypes. Among them, the specimen at U [U0104026], which is complete and well preserved, is designated here as the lectotype, in accordance with Arts. 9.3 and 9.12 of the ICN (Turland et al. 2018).

*Garcinia leucandra* was described by Pierre (1883) based on *Herb. L. Pierre no. 4166* from Sumatra. Two sheets of this original material, labeled “*Treub* (*Herb. L. Pierre no. 4166*),” were located at P [P04700360, P04700362], collected from the same locality. In accordance with Art. 9.6 of the ICN (Turland et al. 2018), these sheets constitute syntypes. Among them, the better-preserved specimen at P [P04700362] is designated here as the lectotype, in accordance with Arts. 9.3 and 9.12 of the ICN (Turland et al. 2018).

##### Notes.

Examination of the type specimens of *G. bancana*, *G. oxyedra*, *G. lamponga*, and *G. leucandra* indicates that they represent the same species, in agreement with Vesque (1893). These four names are lectotypified here.

POWO (2026) lists *G. oxyphylla* Miq., *G. cymulosa* Miq., and *G. hookeri* Pierre as synonyms of *G. bancana*. Examination of the type specimens of *G. oxyphylla* and *G. cymulosa* indicates that they are distinct from *G. bancana*. The type of *G. hookeri* has not been examined.

In Peninsular Malaysia, this species occasionally reaches up to 33 m in height (Whitmore 1973).

Whitmore (1973) reduced *Garcinia curtisii* Ridl. to a variety under *G. bancana* as *G. bancana* var. curtisii (Ridl.) Whitmore, based on the earlier treatment by Ridley (1922). However, based on previous studies (Ridley 1922; Whitmore 1973) and examination of herbarium specimens by Ngernsaengsaruay and Chanton (2026), *G. bancana* var. curtisii cannot be morphologically distinguished from *G. oliveri* Pierre. It is therefore treated as a synonym of *G. oliveri*.

*Garcinia bancana* is distinguished by its leaves elliptic, obovate, or oblanceolate-obovate, 10–23 × 4–11.5 cm, with apex acute, rounded, retuse, or emarginate; stigma, including persistent stigma, unlobed and rough; and fruits globose or subglobose, 3–5 × 2.8–5.5 cm, unlobed, with apex concave or not, and base concave. In contrast, *G. oliveri* has leaves elliptic or oblong-elliptic, 12–25 × (4–)5.5–9 cm, with apex acute or acuminate; stigma, including persistent stigma, radiate, shallowly 6–10-lobed, and papillate; and fruits variable in shape (subglobose, globose, broadly ellipsoid, or sometimes broadly obovoid), 3.5–5 × 3–4.5 cm, sometimes oblique or asymmetrical, unlobed or weakly 6–10-lobed, with a short, thick beak at the apex and base not concave.

##### Additional specimens examined.

**Thailand** • **Peninsular**: Surat Thani [peat swamp forest, Surat Thani Rajabhat University, *Mr Aroon Sinbumroong* personal observation]; • Krabi [Khao Pra-Bang Khram Wildlife Sanctuary, Khlong Thom District, ♂ fl. 4 Apr 1988 (as *Garcinia forbesii*), *C. Niyomdham & W. Ueachirakan 1758* (AAU, BKF, C, K, P [P04701648])]; • Satun [Khlong Ton, 10 Mar 1928 (as *G. cf. benthamii*), *A. F. G. Kerr 14430* (L [L2408706]); • Adang Island, Tarutao National Park, fl., 20 Feb 1981, *G. Congdon 1206* (PSU); • Thale Ban National Park, Khuan Don District, fl., 18 Mar 2004 (as *Garcinia* sp.), *S. Gardner ST0244* (BKF, K)]; • Songkhla [Ton Nga Chang Wildlife Sanctuary, Hat Yai District, fr., 27 Dec 1984, *J. F. Maxwell 84-566* (AAU, BKF, P [P05062477, P04899141], PSU); • ibid., fl., 23 Mar 2004 (as *Garcinia* sp.), *S. Gardner & P. Sidisunthorn ST0293* (K); • ibid., fl. & fr., 24 Mar 2004 (as *Garcinia* sp.), *S. Gardner & P. Sidisunthorn ST0300* (BKF, K); • ibid., fl., 15 May 2004, *S. Gardner & P. Sidisunthorn ST0300*a (BKF); • ibid., fl., 12 May 2004 (as *Garcinia* sp.), *P. Sidisunthorn ST0499* (BKF, K); • ibid., fr., 21 Mar 1993 (as *Diospyros* sp.), *P. Chantaranothai et al. 1279* (K)]; • Yala [Than To Forest, fl., 20 Feb 1969 (as *Garcinia* sp.), *Unknown 2* (BKF [50346])]; • Narathiwat [Phru Khok Ku (originally “Phlu Kok Ku” on the label), Ban Bang Toei, Tak Bai District, fr. 2 Jul 1983 (as *G. cowa*), *C. Niyomdham 576* (BKF, C); • Phru Khok Ku (originally “Plu Kok Kuu” on the label), Tak Bai District, fl., 18 Apr 1986 (as *G. cowa*), *C. Niyomdham et al. 1199* (AAU, BKF, C, K, P [P04701431]); • Su-ngai Padi District, fr., 17 Jul 1984 (as *G. cowa*), *A. Premrasmi 15* (AAU, BKF, C); • Pong Pong Waterfall, fl. buds, 19 Jun 1992 (unidentified), *K. Larsen et al. 43020* (AAU); • Sirindhorn Peat Swamp Forest Reserch Center (“To Daeng” on the label), Su-ngai Kolok District, sterile, 7 Aug 1993, *T. Santisuk et al. s.n*. (BKF [97142]); • Hala-Bala Wildlife Sanctuary, Waeng District, fr., 22 Mar 2000 (as *G. forbesii*), *C. Niyomdham et al. 6120* (AAU, BKF, C); • ibid., fr., 22 Dec 2003 (as *G. atroviridis*), *M. Promchua 67* (CMUB); • ibid., young fr., 26 Aug 2006, *M. Poopath et al. 264* (E [E00375483])]; • Province and locality unspecified [s.d., (as *Garcinia* sp.), *Winit 81* (BKF [8452])].

#### Garcinia costata

Taxon classificationPlantaeMalpighialesClusiaceae

Hemsl. ex King, J. Asiat. Soc. Bengal, Pt. 2, Nat. Hist. 59: 161. 1890

B70FC0D3-3725-5CC6-88E8-013CCD83DEA1

Figs 2B, 4

Garcinia costata Hemsl. ex King, J. Asiat. Soc. Bengal, Pt. 2, Nat. Hist. 59: 161. 1890; Vesque, A. DC. & C. DC., Monogr. Phan. 8: 409. 1893; Ridl., Fl. Malay Penins. 1: 173. 1922; Whitmore in Whitmore, Tree Fl. Malaya 2: 208. 1973; I. M. Turner, Gard. Bull. Singapore 47(1): 261. 1995; S. Gardner et al., Forest Trees S. Thailand 1: 350, fig. 538. 2015. Type. Peninsular Malaysia, Perak, Larut, Maxwell’s Hill, Dec 1833, *G. King’s Collector 5375* (lectotype: designated by Nazre et al. (2018), K! [K000380478] (Fig. 2B); isolectotypes: CAL digital images! [CAL0000005806, CAL0000005809], KEP [reported by Nazre et al. (2018), not seen], L digital image! [L0489715], P digital image! [P00329879], US digital image! [US02961117]).

##### Description.

***Habit*** evergreen trees, dioecious, 8–15(–20) m tall, 30–100 cm GBH; exudate yellow and sticky; young branchlets green, depressed circular in cross section, glabrous. ***Bark*** dark brown, smooth or scaly, with lenticels; inner bark pale red. ***Terminal bud*** concealed between the bases of the uppermost pair of petioles. ***Leaves*** decussate; lamina elliptic, 16–26 × 7–13 cm, apex acute, base cuneate, margin entire or repand, subcoriaceous, dark green above, paler below, glabrous on both surfaces, midrib flattened above, strongly raised below, secondary veins 10–18 on each side, 0.7–1.5 cm apart from each other, departing from the midrib at an angle of 50°–70°, curving towards the margin and connected in distinct loops and united into an intramarginal vein, flattened) above, strongly raised below, intersecondary veins mostly absent, tertiary veins scalariform, veinlets reticulate, visible on both surfaces, interrupted long wavy glandular lines, of differing lengths, nearly parallel to the secondary veins and running to the margin, visible below; petiole green, 1.7–3 cm long, 3–7 mm diam., glabrous, with a basal appendage clasping the branchlet; fresh leaves brittle when crushed. ***Inflorescences*** terminal, borne on short, leafless branchlets, in fascicles of 3–5-flowered cymes in staminates, or a solitary flower in pistillates. ***Flowers*** unisexual, 4-merous; pedicel pale green, glabrous; bracteoles early caducous, triangular, 2–3 mm long; sepals and petals decussate, concave, slightly thick and fleshy, glabrous; sepals pale green; petals white. ***Flower buds*** pale green, subglobose or globose, 0.5–1.2 cm diam. ***Staminate flowers*** 2.5–3 cm diam.; pedicel 0.7–1 cm long, 2–4 mm diam.; sepals 4, suborbicular ca. 7 mm diam., the outer pair slightly larger than the inner pair, apex rounded; petals 4, suborbicular or orbicular, ca. 1.5 cm diam., subequal, larger than sepals, apex rounded; stamens numerous, united into a single central bundle surrounding a pistillode; filaments very short; anthers small, 4-thecous, longitudinally dehiscent; pistillode fungiform. ***Pistillate flowers*** not seen. ***Fruits*** berries green turning pink to red when ripe, depressed globose, 4–5 × 5–8 cm, 8–12-lobed and vertically 8–12-grooved; pericarp coarsely wrinkled when dry; persistent stigma discoid, radiate, 8–12-lobed, rough; with persistent sepals; fruiting stalk short and thick, 3–5 mm long. ***Seeds*** 8–12, sometimes aborted (1–4), with a fleshy pulp.

**Figure 4.**
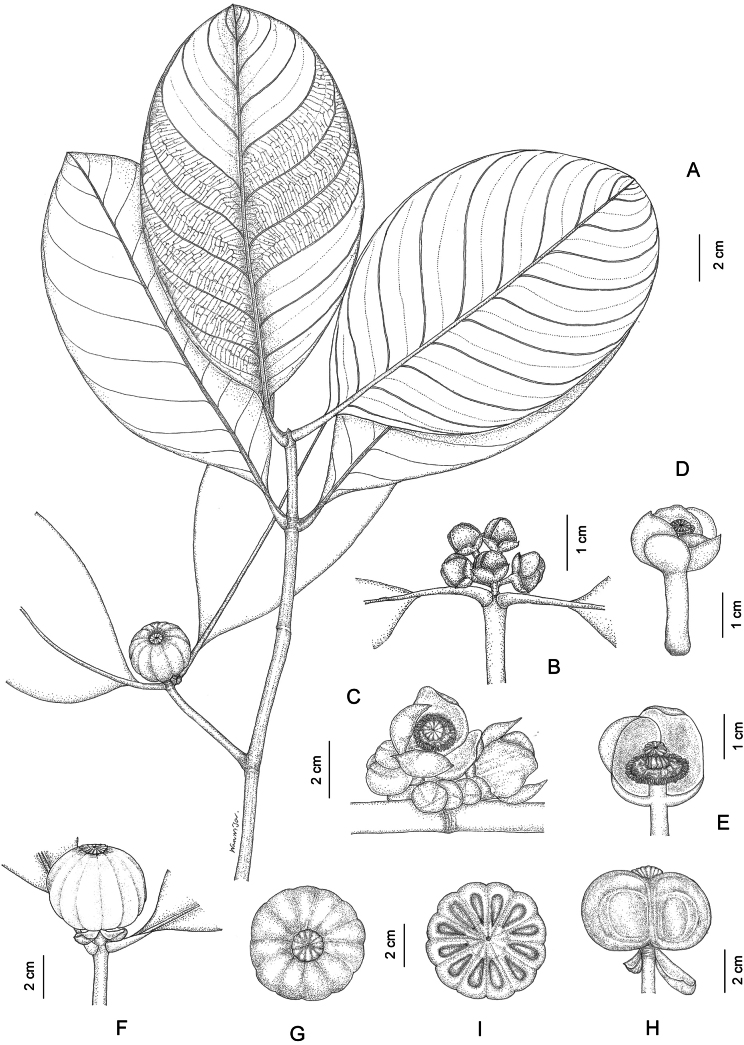
*Garcinia costata*. **A**. Branchlets with leaves and fruit; **B**. Branchlets with inflorescences bearing flower buds; **C**. Branchlets with inflorescences bearing open staminate flowers and flower buds; **D**. Open staminate flower; **E**. Open staminate flower (two petals removed); **F**. Branchlets with fruit; **G**. Fruit (top view); **H**. Fruit (longitudinal section); **I**. Fruit (transverse section). Photo: drawn by Wanwisa Bhuchaisri.

##### Distribution.

Peninsular Thailand, Peninsular Malaysia (Kedah, Perak, and Pahang) (Fig. 3B).

##### Distribution in Thailand.

**Peninsular**: Trang, Satun, Songkhla (Fig. 3B).

##### Habitat and ecology.

The species occurs in tropical lowland evergreen rainforests at elevations of 100–200 m a.s.l.

##### Phenology.

Flowering from December to March; fruiting in March.

##### Conservation status.

*Garcinia costata* has a distribution extending from Peninsular Thailand to Peninsular Malaysia. The species has an EOO of 17,253.89 km^2^ and an AOO of 24 km^2^. In Thailand, it is restricted to the Peninsular region, with an EOO of 496.92 km^2^ and an AOO of 12 km^2^. Given its relatively restricted global distribution, limited number of known localities, and small population size (number of mature individuals), *G. costata* is assessed here as Vulnerable (VU) under criterion D1. For comparison, the World Conservation Monitoring Centre (1998) previously assessed the species as VU under criterion D2.

##### Etymology.

The specific epithet *costata* is derived from Latin, meaning “ribbed” or “with prominent ribs or a midrib” (Stearn 1992; Gledhill 2002), and refers to the conspicuously ribbed leaves, which have a prominent midrib and secondary veins.

##### Vernacular names.

**Mang khut pa** (มังคุดป่า) (Satun, from the specimen *A. F. G. Kerr 14577*).

##### Uses.

Not recorded in Thailand. In Peninsular Malaysia, the fruits are eaten (King 1890; Ridley 1922).

##### Notes.

*Garcinia costata* was named by W. B. Hemsley but not validly published until King (1890), who cited material collected by *G. King’s Collector* and *L. Wray* (without collector numbers) from Perak and deposited in the Herbarium of Kew. Nazre et al. (2018) designated *G. King’s Collector 5375* at K [without barcode] as the lectotype, with isolectotypes at KEP [without barcode] and L [without barcode]. The lectotype was located at K [K000380478], with isolectotypes at CAL [CAL0000005806, CAL0000005809], L [L0489715], P [P00329879], and US [US02961117]. An isolectotype at KEP could not be located.

King (1890) described the petals of this species as pale yellow tinged with red, whereas the specimens examined in the present study have white petals.

Pistillate flowers were not observed in the examined material. However, King (1890) described them as solitary and terminal, borne on short, thick pedicels, with sepals and petals similar to those of the staminate flowers; staminodes ca. 12; an ovary with numerous vertical grooves; and a large, discoid stigma with radiating grooves and a wavy margin.

According to Nazre et al. (2018), *G. costata* is distinguished from species of *Garcinia* sect. *Garcinia* by its large leaves with conspicuous ribs. The staminate flowers resemble those of *G. anomala*, with a mass of stamens surrounding the pistillode. The fruit further differs in having a distinctly lobed or segmented pericarp. Nevertheless, *G. costata* is placed here in *Garcinia* sect. *Brindonia*, following Gaudeul et al. (2024).

##### Additional specimens examined.

**Thailand** • **Peninsular**: Trang [Khao Chong Waterfall, Khao Banthat Wildlife Sanctuary, Na Yong District, fl., 25 Dec 2006, *S. Gardner ST2834* (BKF, K)]; • Satun [Khlong Thon, fr., 14 Mar 1928 (as *Garcinia* sp., *G. cf. plachoni*), *A. F. G. Kerr 14577* (BK, BM, K, L [L2417257]); • Locality unspecified, fl., 16 Mar 1928 (as *G. cf. plachoni*), *M. C. Lakshnakara 350* (BK, K, L [L2417258])]; • Songkhla [Ton Nga Chang Wildlife Sanctuary, Hat Yai District, fl., 22 Mar 2004 (as *Garcinia* sp.), *S. Gardner & P Sidisunthorn ST0277* (BKF, K)].

#### Garcinia parvifolia

Taxon classificationPlantaeMalpighialesClusiaceae

(Miq.) Miq., Ann. Mus. Bot. Lugduno-Batavi 1(7): 208. 1864.

9B2ED29E-EC03-5DE8-8BAE-45490CD72E8D

Figs 5, 6

Garcinia parvifolia (Miq.) Miq., Ann. Mus. Bot. Lugduno-Batavi 1(7): 208. 1864; Pierre, Fl. Forest. Cochinch. 1(5): 30, t. 89F. 1883; Vesque, Epharmosis 2: 22, t. 149, 150. 1889 et in A. DC. & C. DC., Monogr. Phan. 8: 453. 1893; Ridl., J. Straits Branch Roy. Asiat. Soc. 54: 20. 1910 et Fl. Malay Penins. 1: 177. 1922; Corner, Gard. Bull. Straits Settlem. 10(1): 37. 1939 et Wayside Trees Mal. 1: 319, fig. 111. ed. 2. 1952; Corner & Watan., Ill. Guide Trop. Pl.: t. 193. 1969; Backer & Bakh. f., Fl. Java (Spermatoph.) 1: 387. 1963; Kochummen & Whitmore, Gard. Bull. Singapore 26(2): 273. 1973; Whitmore in Whitmore, Tree Fl. Malaya 2: 219. 1973; H. Keng, Concise Fl. Singapore 2: 49. 1990; I. M. Turner, Gard. Bull. Singapore 47(1): 262. 1995; S. Gardner et al., Forest Trees of S. Thailand 1 (A–Es): 360. fig. 550. 2015.
≡ Rhinostigma parvifolium Miq., Fl. Ned. Ind., Eerste Bijv. 3: 495. 1861. Type. Indonesia, Sumatra, Priaman, s.d., *Diepenhorst HB2093* (lectotype: designated here, U digital image! [U0002407]; isolectotype: L digital image! [L2417013]).
*= Garcinia globulosa* Ridl., J. Straits Branch Roy. Asiat. Soc. 54: 20. 1910; Fl. Malay Penins. 1: 176. 1922. Type. Singapore, Garden Jungle, Apr 1898, *Ridley 9195* (lectotype: designated here, SING! [SING0063129]).

##### Description.

***Habit*** evergreen trees, dioecious, 10–30 m tall, 50–200 cm GBH, buttressed near the base of the stem in large trees; exudate cream or yellow and sticky; branches decussate, horizontal or nearly horizontal; young branchlets green, 4-angular, glabrous. ***Bark*** dark brown, scaly; inner bark red. ***Terminal bud*** concealed between the bases of the uppermost pair of petioles. ***Leaves*** decussate; lamina elliptic or narrowly elliptic, 5–13 × 2–4.5 cm, apex tapering to a long blunt tip, 1–2.5 cm long, base cuneate, margin entire or repand, subcoriaceous, glossy dark green above, paler below, glabrous on both surfaces, midrib shallowly grooved above, raised below, secondary veins 7–11 on each side, curving towards the margin and connected in distinct loops and united into an intramarginal vein, flattened above, slightly raised below, with intersecondary veins, veinlets reticulate, visible above, faint below, scattered black gland dots and interrupted long wavy glandular lines, of differing lengths, running across the secondary veins to the apex, visible below; petiole green, 0.5–1 cm long, 1.5–3.5 mm diam., grooved above, glabrous, with a basal appendage clasping the branchlet; fresh leaves brittle when crushed; mature leaves turning greenish yellow to yellow before falling off. ***Inflorescences*** terminal or axillary, borne on short, leafless lateral branchlets, in fascicles of 4–12-flowered cymes (staminate inflorescence usually bearing more flowers than pistillate ones). ***Flowers*** unisexual, 4-merous; pedicel pale green to greenish yellow or yellow, glabrous; bracteoles early caducous, triangular, 2–5 × 1–2 mm long; sepals and petals decussate, concave, pale yellow to yellow, slightly thick and fleshy, glabrous. ***Flower buds*** pale green, becoming greenish yellow to yellow before anthesis, subglobose or globose, 2–3 mm diam. ***Staminate flowers*** 0.5–1 cm diam., usually smaller than pistillate ones; pedicel 4–7 mm long, 1–1.5 mm diam.; sepals 4, suborbicular or broadly elliptic, 3–4 × 2.5–4 mm, the outer pair slightly larger than the inner pair, apex rounded; petals 4, broadly obovate, 4–6 × 3–5.5 mm, subequal, apex rounded; stamens numerous, united into a single central 4-sided bundle; filaments very short; anthers small, 4-thecous, longitudinally dehiscent; pistillode absent. ***Pistillate flowers*** 1–1.2 cm diam. (slightly larger than staminate ones); pedicel 2–3 mm long, 2.5–3 mm diam., usually shorter and thicker than staminate ones; sepals and petals same as in staminate flowers; staminodes absent; pistil fungiform; ovary pale green, subglobose, 2–3.5 × 3–4 mm; stigma unlobed, rough. ***Fruits*** berries, green turning yellow to orangish yellow, orange, orangish red, or red when ripe, smooth and glabrous, depressed globose or subglobose, 2–3 × 1.5–3 cm, unlobed, apex and base concave, pericarp fleshy; persistent stigma more or less sunken, 2–3 mm diam., unlobed, rough; persistent sepals slightly larger than in flowering material; fruiting stalk short and thick, 3–5 mm long, 3–3.5 mm diam. ***Seeds*** 2–5, sometimes aborted, semi-ellipsoid, 7–9 × 4–6.5 mm, rounded at both ends, with a white fleshy pulp.

**Figure 5.**
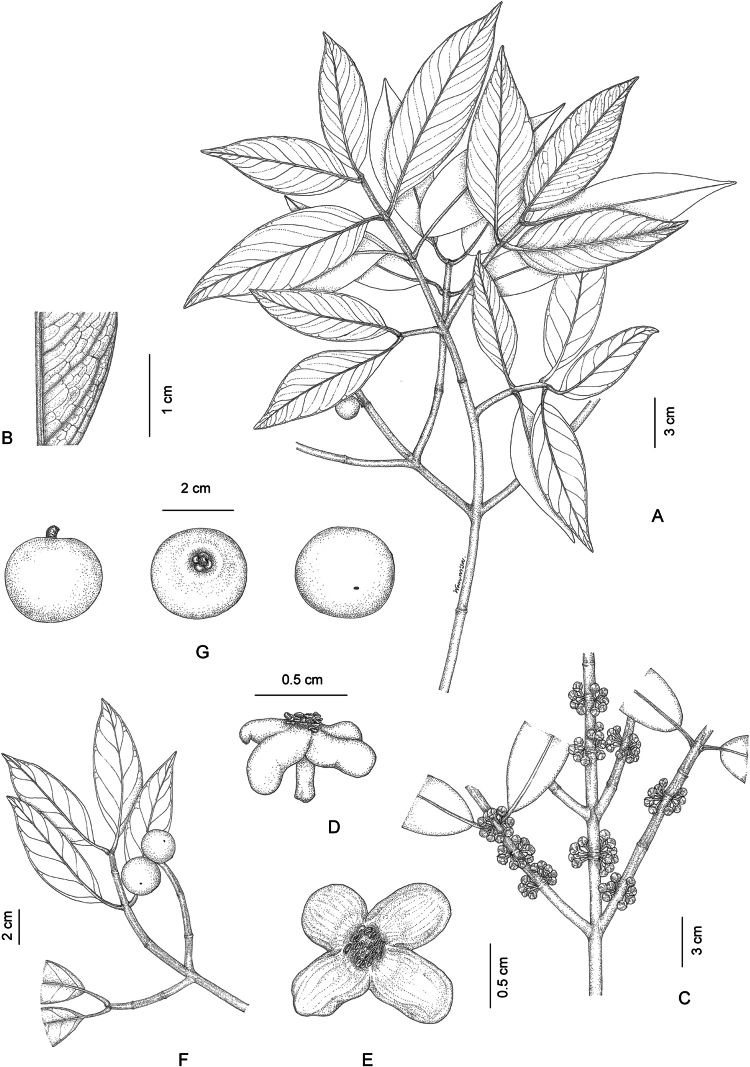
*Garcinia parvifolia*. **A, F**. Branchlets with leaves and fruits; **B**. Part of the abaxial surface of the leaf; **C**. Branchlets with inflorescence bearing flower buds; **D**. Open staminate flower (side view); **E**. Open staminate flower (top view); **G**. Fruits. Photo: drawn by Wanwisa Bhuchaisri.

**Figure 6.**
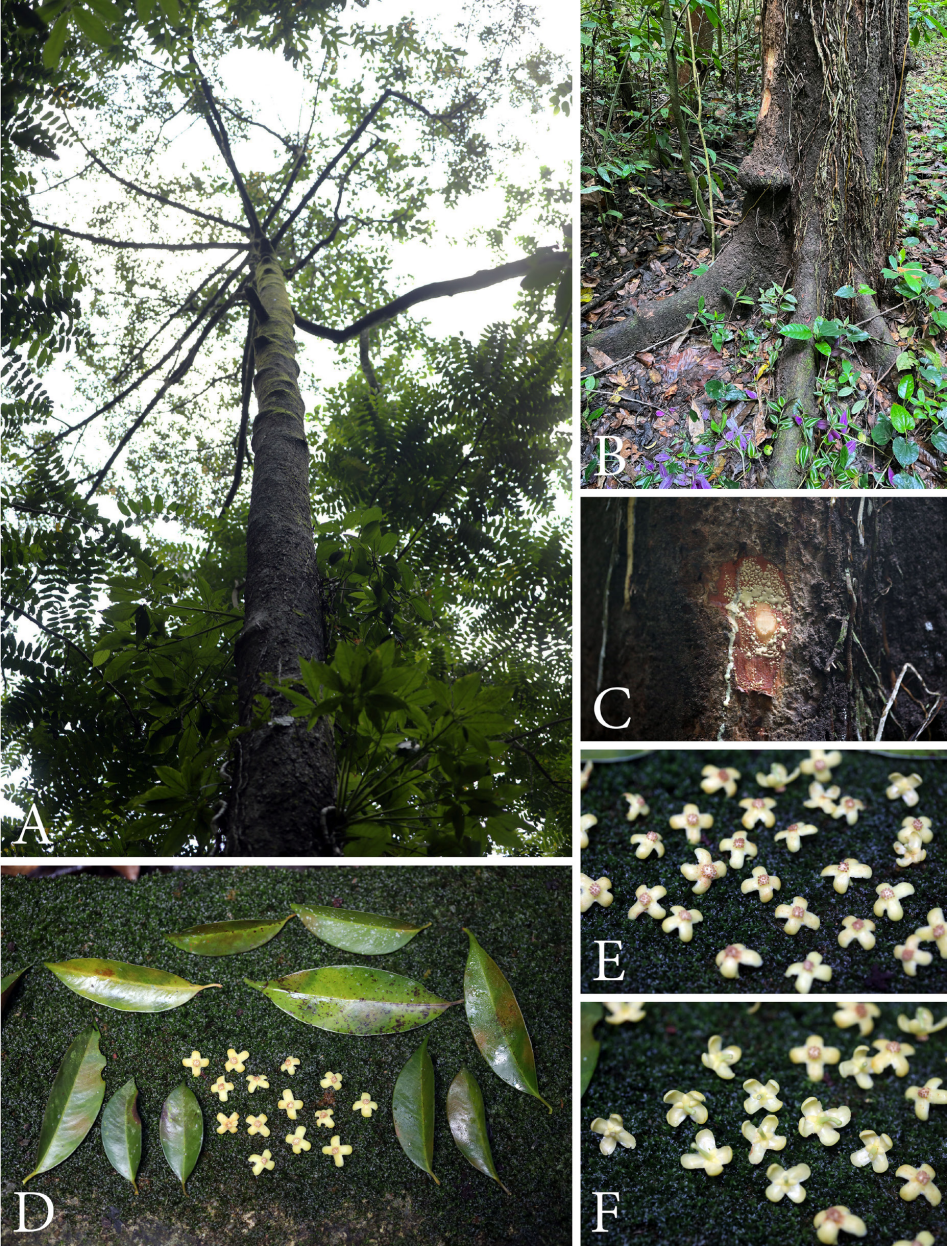
*Garcinia parvifolia*. **A**. Habit; **B**. Buttressed near the base of the stem in a large tree; **C**. Slashed bark with cream exudate; **D**. Leaves and staminate flowers; **E, F**. Staminate flowers. Photos: Chatchai Ngernsaengsaruay.

##### Distribution.

Peninsular Thailand, Peninsular Malaysia (Kedah, Penang, Perak, Pahang, Terengganu, Selangor, Negeri Sembilan, and Johor), Singapore, Indonesia (Sumatra, Java, Lesser Sunda Islands, Sulawesi, and Maluku), Borneo (Sabah, Sarawak, Brunei, East Kalimantan, West Kalimantan, and South Kalimantan), New Guinea (Fig. 3C).

##### Distribution in Thailand.

**Peninsular**: Ranong, Surat Thani, Phangnga, Krabi, Nakhon Si Thammarat, Trang, Satun, Songkhla, and Narathiwat (Fig. 3C).

##### Habitat and ecology.

*Garcinia parvifolia* occurs in tropical lowland evergreen rainforests, sometimes along streams, with or without bamboo, at elevations from near sea level to 350 m a.s.l. Niyomdham (1997) reported that the species occurs in peat swamp forests of Narathiwat Province; however, no specimens from this forest type were seen for the present study. In Ayer Hitam Forest Reserve, Johor, Peninsular Malaysia, it has been recorded from peat swamp forests (Whitmore 1973).

##### Phenology.

Flowering from February to May; fruiting from April to July.

##### Conservation status.

*Garcinia parvifolia* has a distribution extending from Peninsular Thailand to Borneo and New Guinea. Globally, the species has an EOO of 7,560,436.72 km^2^ and an AOO of 836 km^2^. In Thailand, it is confined to the Peninsular region, with an EOO of 58,159.96 km^2^ and an AOO of 60 km^2^. Given its wide geographic distribution, occurrence in many localities, and the absence of any known or immediate threats to the species or its habitats, *G. parvifolia* is here assessed as LC, in agreement with Zainal Ariffin (2023).

##### Etymology.

The specific epithet *parvifolia* is derived from the Latin compound words *parvi*- meaning small and -*folia* meaning -leaved (Radcliffe-Smith 1998; Gledhill 2002), referring to the small leaves of the species.

##### Vernacular names.

**Cha muang lek** (ชะมวงเล็ก) (Narathiwat); Muang (มวง) (Surat Thani, Nakhon Si Thammarat) (from *A. F. G. Kerr 12464, 15643*); Muang sai (มวงทราย) (Trang) (from *S. Phusomsaeng 163*); Ka-le-bu-ko (กาเละบูโกะ) (Malay-Songkhla) (from *A. F. G. Kerr 14856*); Kandis, Wild yellow kandis (Corner 1952; Corner and Watanabe 1969); Kandies (Vesque 1893).

##### Uses.

The fruits are edible and sour. In Malaysia and Sumatra, they are used as a sour flavoring (Bircher and Bircher 2000). In Singapore and Sumatra, the relatively hard wood is used for indoor construction (Burkill et al. 1966).

##### Lectotypifications.

*Rhinostigma parvifolium* was described by Miquel (1861) and later transferred to *Garcinia* by Miquel (1864), who cited collections from Sumatra: *D.* (*Diepenhorst*) from “Priaman” and *T.* (*Teysmann*) from “Bangka, prope Djebus,” without indicating the herbaria in which the specimens were deposited. Specimens of *Teijsmann HB3270* (“*HB3270*” on the label) collected from Bangka, Jeboes, were located at BO [BO0116825], L [L2417008], MEL [MEL2436671, MEL2436672], and U [U0226790]; *Diepenhorst HB2093* from Priaman at L [L2417013] and U [U0002407]; and *Diepenhorst HB2212* from Priaman at BO [BO0116826], L [L2417009], and U [U1199416]. According to Art. 9.6 of the ICN (Turland et al. 2018), these specimens constitute syntypes. Among them, the specimen *Diepenhorst HB2093* at U [U0002407], which forms part of Miquel’s private herbarium, is here designated as the lectotype, in accordance with Arts. 9.3 and 9.12 of the ICN (Turland et al. 2018).

Ridley (1910) described *Garcinia globulosa* based on syntypes from Singapore [*Ridley 9195* from Garden Jungle; *Ridley 4450, 9142* from Bukit Timah; *Ridley 266, 1825, 1966, 1968* from Selitar; *Hullett 41* from Alma and Changi], Perak [*Wray 2531, 3183* from Batu Togoh], Selangor [*King’s Collector 8539* from near Ulu Selangor], and Malacca [*Goodenough 1270* from Bukit Beruang; *Cantley s.n*. from Selangor]. Of these, only three gatherings were located: *Ridley 9195* at SING [SING0063129]; Wray 2531 at K [K000677664] and SING [three sheets without barcodes]; and *King’s Collector 8539* at P [P04701606]. Among them, the specimen *Ridley 9195* at SING [SING0063129], which is well preserved and clearly shows the diagnostic characters of the species, is here designated as the lectotype, in accordance with Arts. 9.3 and 9.12 of the ICN (Turland et al. 2018).

##### Additional specimens examined.

**Thailand** • **Peninsular**: Ranong [Khlong Kamphuan, Suk Samran District, fl., 5 Feb 1929 (as *Garcinia* sp.), *A. F. G. Kerr 17025* (BM, C, K, P [P05062039])]; • Surat Thani [Khao Nuo (originally “Kao Nuo” on the label), fl., 28 Mar 1927 (as *G. merguensis*), *A. F. G. Kerr 12464* (BM, K)]; • Phangnga [Khao Lak Lam Ru National Park, a few km West of Ton Chong Fa Waterfall, Takua Pa District, fr., 12 Jun 2004 (as *Garcinia* sp.), *S. Gardner & P. Sidisunthorn ST0720* (BKF, K)]; • Krabi [Than Bok Khorani National Park, Chatchai Ngernsaengsaruay personal observation with photos]; • Nakhon Si Thammarat [Ban Nathon (originally “Ban Natawn” on the label), fl., 10 May 1928 (as *Garcinia* sp.), *A. F. G. Kerr 15643* (BM, K); • Khao Luang National Park, Karome Waterfall, Lan Saka District, ♂ fl., 18 Mar 1985, *J. F. Maxwell 85-318* (AAU, BKF, E [E00160905, E00839793], L [L2417046], P [P04701877], PSU)]; • Trang [Khao Chong, fl. & fr., 16 Apr 1969 (as *Garcinia* sp.), *S. Phusomsaeng 163* (BKF, C); • Khao Chong, fr., 14 Jul 1985, *J. F. Maxwell 85-721* (AAU, BKF, L [L2417045], PSU); • Khao Chong, Na Yong District, fr., 8 Jul 2000 (as *Garcinia* sp.), *D. J. Middleton et al. 317* (BKF); • Khao Chong 24-hectare plot, fr., 2003 (as *Garcinia* sp.), *A. Sinbumroong & S. Davies AS428* (BKF); • Peninsular Botanic Garden (Thung Khai), Yan Ta Khao District, ♂ fl., 2 Mar 2004 (as *Garcinia* sp.), *S. Gardner & P. Sidisunthorn ST0061* (BKF, K); • ibid., fl., 5 Mar 2004 (as *G. bancana*), *S. Gardner & P. Sidisunthorn ST0172* (BKF, K)]; • Satun [Tarutao National Park, Ao Makham, La Ngu District, fl., 17 Feb 2005 (as *Garcinia* sp.), *S. Gardner & P. Tippayasri ST1548* (BKF, K); • ibid., fl., 28 Mar 2006 (as *Garcinia* sp.), *S. Gardner ST2521* (BKF, K)]; • Songkhla [ originally “Ban Pen” on the label], ♂ fl., 28 Mar 1928 (as *G. merguensis*), *A. F. G. Kerr 14856* (BM, C, K); • Ton Nga Chang Wildlife Sanctuary, Hat Yai District, ♂ fl., 24 Mar 2004 (as *Garcinia* sp.), *S. Gardner & P. Sidisunthorn ST0307* (BKF, K); • Pa Krat Non-Hunting Area, Na Thawi District, fl., 3 Mar 2005 (as *Garcinia* sp.), *S. Gardner & P. Sidisunthorn ST1588* (BKF, K)]; • Narathiwat [Hala-Bala Wildlife Sanctuary, Waeng District, fr., 9 Jun 2002 (as *Garcinia* sp.), *P. Puudjaa 1090* (BKF); • ibid., ♂ fl., 18 Mar 2009 (as *G. merguensis*), *C. Niyomdham & P. Puudjaa 8461* (BKF)].

## Supplementary Material

XML Treatment for Garcinia bancana

XML Treatment for Garcinia costata

XML Treatment for Garcinia parvifolia

## Additional information

### Artificial Intelligence (AI) use

The authors accept full responsibility for the content of the manuscript, including the disclosure of any use of AI.

No AI tools were used in the preparation of this manuscript.

### Author ORCIDs

C. Ngernsaengsaruay https://orcid.org/0000-0002-7131-976X

### Data availability

All of the data that support the findings of this study are available in the main text.
